# High-quality complete genome sequence of *Xanthomonas oryzae* pv. *oryzicola* (*Xoc*) strain HB8

**DOI:** 10.1128/MRA.00459-23

**Published:** 2023-08-01

**Authors:** Wei Yang, Xingxun Liu, Meng Liu, Fengmei Wei, Lei Yang, Meng Yuan, Guotian Li

**Affiliations:** 1 National Key Laboratory of Agricultural Microbiology, Hubei Hongshan Laboratory, Hubei Key Laboratory of Plant Pathology, The Center of Crop Nanobiotechnology, Huazhong Agricultural University, Wuhan, Hubei, China; 2 National Key Laboratory of Crop Genetic Improvement, Huazhong Agricultural University, Wuhan, Hubei, China; University of Arizona, Tucson, Arizona, USA

**Keywords:** *Oryza sativa*, *Xanthomonas*, nanopore, TALE genes, genome

## Abstract

Here, we report a high-quality genome of *Xanthomonas oryzae* pv. *oryzicola* (*Xoc*) strain HB8, which causes bacterial leaf streaks in rice. The genome size of HB8 is 4,800,100 bp, with a GC content of 64.03%, which serves as an important resource for the study of the *Xanthomonas*-rice pathosystem.

## ANNOUNCEMENT

Bacterial leaf streak (BLS), caused by *Xanthomonas oryzae* pv. *oryzicola* (*Xoc*), is a devastating bacterial disease in rice, primarily in South China and Southeast Asia (1, 2). Even so, it poses a more serious threat to rice than the bacterial leaf blight caused by *Xanthomonas oryzae* pv. *oryzae* (*Xoo*) (3). *Xoc* was first reported in the Philippines in 1918 (4). Upon entering rice leaves through wounds or stomata, *Xoc* colonizes the interstices of mesophyll cells, resulting in leaf lesions (5) and yield losses of up to 32% (6). Considering its high virulence toward Zhonghua 11 and other frequently used rice varieties, we sequenced and assembled the complete genome of *Xoc* strain HB8, which serves as a valuable resource for analyzing bacterial pathogenesis and interactions in the *Xanthomonas*-rice pathosystem.

The *Xoc* strain HB8 was isolated from infected rice leaves in Hubei Province, China (7). In infection assays, HB8 causes typical leaf streak symptoms in rice. The sample was cultured on nutrient broth plates for 36 hr with aeration (180 rpm) at 28°C under aerobic conditions. After selecting a single colony from the culture, it was mixed with 50% glycerol and stored at −80°C until use. During the original isolation and culture process, the sample underwent five passages.

High molecular weight genomic DNA was extracted from a single colony and prepared using the QIAGEN Genomic Kit. For Nanopore sequencing, the library was constructed using the ligation sequencing kit (SQK-LSK109) without shearing and sequenced on the PromethION flow cells (FLO-PRO002). Using Guppy v6.2.1 (8) rapid base calling with concurrent barcode trimming, we obtained a total of 51,986 long reads with an N50 of 35,701 bp. The short reads library was prepared using the MGIEasy RNA Library Prep Set from the DNBSEQ-T7 platform to generate 150 bp paired-end reads. The final short reads data contained 6,966,855 reads after filtering low-quality reads using Fastp v0.23.2 with default parameters (9).

**Fig 1:**
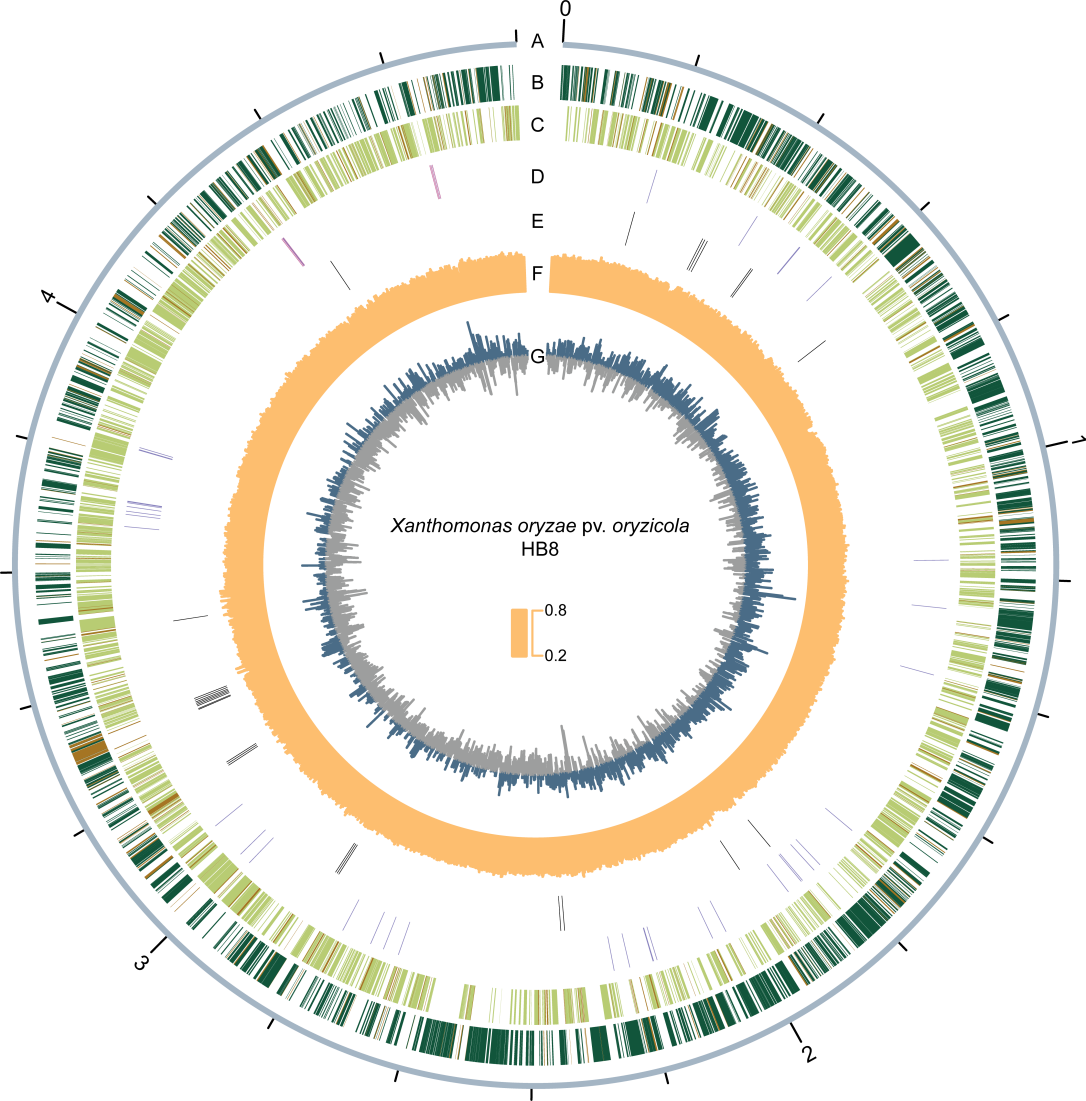
Graphical representation of the genome map of *Xanthomonas oryzae* pv. *oryzicola* (*Xoc*) strain HB8. (**A**) Genome size (Mb). (**B**) Predicted CDSs transcribed in the clockwise direction: protein-coding genes (green) and pseudogenes (brown). (**C**) Predicted CDSs transcribed in the counterclockwise direction: protein-coding genes (green) and pseudogenes (brown). (**D**) rRNA (pink) and tRNA (purple). (**E**) Predicted TALE encoding genes. (**F**) GC content. (**G**) GC skew. Data in circles are displayed in nonoverlapping 1,000 bp intervals.

High-quality DNA was extracted to identify the 16S RNA region as described (10). Finally, the strain HB8 was highly similar (100%) to *Xoc* using BLAST (E-value < 1e-5) with the NCBI standard databases (accession no: CP045912.1). Default parameters were used for all software unless otherwise stated, and we used Unicycler v0.5.0 to assemble the HB8 genome (11). The final HB8 genome consists of a single circular contig at 4,800,100 bp, with a GC content of 64.03%. Using BUSCO v5.4.4, we observed that the HB8 genome covers 100% of the genes in the bacteria_odb10 lineage (12). Additionally, we predicted 4,163 genes (3,652 coding and 511 pseudogenes), 53 tRNAs, and 6 rRNAs in the HB8 genome using the Prokaryotic Genome Annotation Pipeline v6.5 (13). Transcription activator-like effector (TALE) genes often act as virulence factors that are critical in the *Xanthomonas*-rice pathosystem (14). A total of 27 TALE genes were annotated using AnnoTALE v1.5 (15). We performed visualization of the genome map of strain HB8 using TBtools v1.120 (16) (Fig. 1). Our work provides a new resource to study *Xanthomonas*-rice interactions and facilitates ongoing efforts to combat BLS.

## Data Availability

The HB8 genome project is available at NCBI under the accession number PRJNA977082. The HB8 genome sequence file is deposited at GenBank under the accession number CP126568. The sequencing raw reads also are deposited under the accession numbers SRR24905655 and SRR24905656 at the SRA database.
